# Efficacy of Electromyographic Biofeedback in Muscle Recovery after Meniscectomy in Soccer Players

**DOI:** 10.3390/s22114024

**Published:** 2022-05-26

**Authors:** Verónica Morales-Sánchez, Coral Falcó, Antonio Hernández-Mendo, Rafael E. Reigal

**Affiliations:** 1 Department of Social Psychology, Social Anthropology, Social Work and Social Services, University of Málaga, 29071 Málaga, Spain; vomorales@uma.es (V.M.-S.); mendo@uma.es (A.H.-M.); 2 Department of Sport, Food and Natural Sciences, Western Norway University of Applied Sciences, 5020 Bergen, Norway; coral.falco@hvl.no

**Keywords:** electromyographic biofeedback, rehabilitation, neuromuscular rehabilitation, sport

## Abstract

Electromyographic biofeedback (EMG-BF) is a therapeutic technique that has been used successfully in the rehabilitation of injuries. Although it has been applied to athletes, its use in this field is not very widespread. The objective of this study is to analyze its effectiveness in the recovery of electromyographic activity of the quadriceps after meniscectomy, evaluated through isometric contraction of the vastus lateralis. The sample comprised ten professional footballers in the Spanish League (2nd Division A) who had previously suffered a meniscus injury in their knee and had undergone a meniscectomy. The intervention consisted of EMG-BF treatment lasting between 6 and 10 sessions. The electromyographic signal was recorded using a Thought Technology ProComp Infiniti 8-channel biofeedback unit with a sampling rate of 2048 samples/second. For each session, a within-subject ABA design of 6 or 10 trials per session was used, with three pre- and three post-measures, which determined the gain for each session. The results indicated (1) improvements in all cases, (2) EMG-BF was effective, (3) the working model was statistically significant with an explained variance of between 67% and 75%, and (4) the generalizability analysis showed that the results are reliable and generalizable. The results indicate that EMG-BF is effective in neuromuscular rehabilitation after this type of intervention.

## 1. Introduction

Biofeedback (BF) is a technique that makes it possible to obtain information on the functioning of a person’s body [1]. Through electronic procedures, physiological signals are transformed into data that can be interpreted using display interfaces. These mechanisms are very useful for improving understanding of the behavior of some organ or system in the human body, as well as learning to regulate its functioning more appropriately [2]. In recent decades, BF has been widely used in various fields and is especially useful in contexts of learning and medical rehabilitation [3,4].

Electromyography (EMG), in turn, is a technique that makes it possible to record the electrical activity of a muscle [5]. Two procedures are commonly used: a needle electrode inserted into the muscle or surface electrodes attached to the muscle. Both detect the electrical signals that the muscle emits when it contracts, which are transmitted to a device that transforms them, usually, into a visual or acoustic signal [6]. Thus, when the muscle is very active, this signal becomes more intense and evident, decreasing when the muscle relaxes. A person undergoing EMG can therefore discover in real time what kind of electromyographic activity is associated with the contraction performed by a specific muscle, becoming familiarized, and learning the sensations associated, with a given level of excitation [7].

Electromyographic biofeedback (EMG-BF) is therefore a non-invasive self-regulation technique using surface electrodes to monitor EMG responses, and operating through the constant feedback or information that the subject receives about the EMG response that they wish to subject to voluntary control [8]. An interesting aspect of EMG-BF is that it enables the person to interact with the EMG unit, on the basis of the information being monitored and the needs of the intervention [2,6]. In this way, tone can be voluntarily regulated according to the objectives being pursued. It has been observed that this technique contributes to exercising greater control over voluntary activation of the muscle and acquiring greater awareness of it [9].

EMG-BF is a technique that has demonstrated its ability to restore muscle control and rehabilitate movement patterns after pathologies such as muscle atrophy, muscle spasticity after a stroke, or sports injuries [10,11,12,13]. It provides information that is difficult to access by natural means, facilitating the voluntary activity of the muscles and also improving the regulation of their involuntary activity [14], which is beneficial in the clinical context. For this reason, its use has been verified, and it has a promising future, in musculoskeletal and neurological rehabilitation [15]. Specifically, it has shown positive effects in patients who have suffered ligament injuries, fractures, or muscle pain caused by various pathologies [16,17,18,19,20,21].

Furthermore, in the context of sports, and specifically soccer, bone, joint, and muscle injuries are very common [22,23]. In this sport, the lower extremities are exposed to greater instability and have to withstand great exertion, so the prevalence of lesions in ankles and knees, as well as in the adjacent muscles, is higher [24,25]. Whether lower limb injuries involve the ligaments, bones, or muscles, a period of inactivity can lead to loss of muscle mass and tone, as well as a loss of ability to regulate muscle contraction, impacting on footballers’ recovery and adaptation to playing [26,27]. In this regard, the scientific literature has shown that EMG-BF can be a useful procedure in the treatment of injuries affecting these structures in the musculoskeletal system, improving the prospects of functional recovery [19,28].

The knee, specifically, is a structurally complex joint and can therefore display a wide variety of clinical situations associated with traumatic events that it may be subjected to [29,30,31]. One of the classic scenarios that can emerge while playing soccer is a meniscal lesion [32,33,34], which may present with varying degrees of severity depending on the damage caused to its structure and whether it occurs in the medial or the lateral meniscus [35]. There is often a substantial alteration in the structure of the meniscus requiring surgical intervention, and it is one of the most common traumatological procedures in the general population [36], specifically in soccer [33,37].

One of the surgical operations commonly used to resolve damage to the meniscus is meniscectomy, a procedure by which a torn meniscus is partially or wholly removed. The object is to reduce the pain caused by the lesion, restore the functional capacity of the knee, and reduce the risk of degenerative osteoarthritis [38]. Formerly, total meniscectomy was the most common procedure; although, this produced greater long-term damage to the joint by increasing its structural instability [38,39]. With the emergence of arthroscopic techniques, there has been an increase in interventions in which only the defective part of the meniscus is removed, so that the insult to the meniscus is limited and the stability of the knee is less compromised [40].

However, despite being less radical, partial meniscectomy has consequences for athletes; in general, because it is a lesion that forces them to temporarily suspend their training process and match fitness, and in particular, because it affects the biomechanical performance of the joint and obliges the athlete to readjust [41,42]. Consequently, the muscles associated with the knee may undergo changes in their structure and functionality, reducing the precision with which they worked up till then. Loss of muscle mass or small variations in postural aspects may result in less effectiveness in their actions. In addition, this could give rise to a loss of confidence, affecting the athlete’s performance [43,44,45,46]. For these reasons, one of the objectives, when athletes are injured, is to try to restore their biomechanical capabilities and musculoskeletal functionality. Among the existing procedures to aid this process of recovery, specifically at the muscular level, is EMG-BF. This technique has been observed to be useful for maintaining and recovering muscle control ability, regulating its functionality and degree of tension [20].

An essential issue when biofeedback procedures are applied is to show that it has a positive impact on restoring functional ability, beyond what is achieved with conventional physical therapy based on performing conventional isometric contraction exercises. The main purpose of this research is therefore to explore the efficacy and effectiveness of EMG-BF treatment in a group of footballers who had suffered a partial tear of the meniscus and had undergone a surgical meniscectomy procedure. To achieve this objective, we present a multiple-case study that aims to comply with the efficacy criteria described by Chambless and Hollon [47]. In all cases, the goal it is hoped to attain is functional ability similar to that of the uninjured contralateral limb.

## 2. Materials and Methods

### 2.1. Design

The design used in this study followed a manipulative strategy of a quasi-experimental within-subject type, with non-random assignment and pretest–posttest (A→B→A) baseline measures. During the pretest and posttest, the participants performed three trials without feedback (phases A and C). Between the pretest and the posttest (phase B), they performed between six and ten tests with electromyographic feedback. The study protocol was registered at https://www.clinicaltrials.gov/ (accessed on 29 April 2022), with the identifier NCT05376072.

Various protocols are used when applying biofeedback. One of them, which is applied in this work, is called BFB (biofeedback) training [48]. It is a method in which a person modifies their muscle activity in a self-controlled way, based on the visualization of recorded signals.

### 2.2. Participants

The participants in this study were ten professional footballers from the second division of the Spanish Football League, aged between 24 and 35 years (M = 29.10; SD = 3.54), all male. Of these players, 30% were left-footed and 70% right-footed. All of them had suffered a partial tear of the meniscus in their dominant leg (3 in the left leg and 7 in the right). All of them had undergone a meniscectomy before the study (in a range of 4 to 7 weeks). Prior to the EMG-BF treatment, the players participated in rehabilitation sessions that were prescribed by the medical services of their clubs and performed functional retraining exercises. In parallel with the EMG-BF sessions, they continued to perform orthopedic rehabilitation exercises. All the participants signed an informed consent form to be able to take part and the principles of the Declaration of Helsinki were respected at all times [49]. The study was also approved by the ethics committee (CEUMA, no. 243, 19-2015-H) of the University of Malaga (Spain). The inclusion criteria were having recently suffered an injury in the right or left knee causing a partial tear of the meniscus and having undergone a surgical intervention with a meniscectomy procedure.

### 2.3. Measurements and Instruments

The electromyographic information was recorded using a Thought Technology ProComp Infiniti 8-channel biofeedback device, which has a sampling rate of between 256 and 2048 samples/second. It is composed of a Decoder Unit and a TT-USB Interface Unit connected by a fiber optic cable. Electrodes of a type placed in a MyoScan-Pro unit and individual electrodes were used. The electrodes were positioned following the anatomical distribution of the muscle.

### 2.4. Procedure

The players performed 10 work sessions with EMG-BF. Each session was divided into three phases: (a) three trials without receiving feedback, (b) between six and ten trials with feedback of electromyographic activity, and (c) three trials without feedback. In each trial, isometric contractions of the vastus lateralis were performed. For all sessions, each isometric contraction lasted six seconds and the participants rested for two minutes between trials to recover their contraction capacity. The differences between phase (A) and phase (C) were analyzed and the electromyographic gains were explored as the sessions were performed.

During maximum effort isometric contraction, the software associated with the ProComp Infiniti biofeedback unit collects the amplitude, the mean and maximum electromyographic signal, and also the contraction and tightening times. The contraction time comprises the interval between the start of the contraction and the achievement of the desired muscle tension. The tightening time is the period in which the tension voluntarily reached is maintained [50]. For this study, the maximum and mean electromyographic activity values were considered, calculated during the muscle tension time, and maintained during each trial.

In all the trials the participants remained in a sitting position, with their legs resting on a chair and both knees extended. There is a relationship between the angle of the knee joint and the maximum electromyographic activity of the quadriceps [51], which is greater when the knee is in full extension.

### 2.5. Data Analysis

The data were subjected to descriptive and inferential analyses. To calculate the descriptive statistics and perform the analysis of variance (ANOVA), SPSS Statistics v.24 (IBM Corp., Armonk, NY, USA) was used. The variance component analysis was performed with SAS v.9.1 (SAS Institute Inc., Cary, NC, USA) [52,53], and for the generalizability analysis, SAGT v.1.0 (University of Malaga, Malaga, Spain) was used [54].

## 3. Results

### 3.1. Variance Component Analysis

A variance component analysis was carried out (Table 1) using a six-facet model [y/z = p l s t b n], both for the maximum EMG signal (y) and for the mean EMG signal (z), where p (participant) × l (laterality) × s (session) × t (trial_type) × b (baseline-trial-baseline) × n (trial_number) were structured in a cross-facet design. These analyses estimate the variance explained in the proposed model by each of the facets of which it is composed and the interaction between them. They enable us to establish to what extent certain study variables determine the changes in others; in this case, the maximum and mean contractile tension generated by the vastus lateralis. A minimum squares strategy (VARCOM TYPE I) and a maximum verosimilitude strategy (GLM) were used. Owing to the saturation produced by working with so many facets, the model [y/z= p l s t b n] was initially used without interactions. It was obtained that the error variance with both procedures was the same (in the y model = p l s t b, the values were GLM = 5,478,311.77/VARCOMP = 5,478,312; in the z model = p l s t b n, the values were GLM = 2,362,841.123/VARCOMP = 2,362,841) and both models proved to be significant with an explained variance of 67.32% and 62.50%, respectively. Moreover, all facets proved to be significant. To obtain a non-saturated model we eliminated the l(laterality) and s(session) facets, which were those that contributed the least variability to the model. As in the previous case, the error variance was the same with both procedures (in the y model = p t b, the values were GLM = 4,114,971.91/VARCOMP = 4,114,972; in the z model = p t b n, the values were GLM = 2,048,639.209/VARCOMP = 2,048,639) and both models proved to be significant, with an explained variance of 75.46% and 67.49%, respectively. Moreover, all the facets individually (p, t, b, and n) and the interactions (p*t), (p*b), and (p*n) were significant in both models. The results obtained in the error variance, with a minimum squares strategy (VARCOM TYPE I) and a maximum verosimilitude strategy (GLM), allow us to assume that the sample is linear, normal, and homoscedastic [55,56].

### 3.2. Generalizability Analysis

With these results, a generalizability analysis was performed and the G indices were estimated in all the possible models (Table 2). These analyses estimate the possibility of generalizing the results found. The relative G index refers to the reliability of the data and the absolute G index to generalizability.

In all the models estimated both for the means and for the maximum, the G indices were between 0.728 and 0.996. Only in two models ([p] [b] [n]/[t] and [b] [n]/[p] [t]) was the absolute G index below 0.5; this was determined by the fact that the [t] facet was the one that showed the most explained variance in all the analyses (Maximum 59.109% and Means 34.885%) (see Table 1). It is important to point out that in the [t] [b] [n]/[p] model, where the number of participants is estimated from t (trial_type) × b (baseline-trial-baseline2) × n (trial_number), the G indices, both relative and absolute, were above 0.97. 

### 3.3. Trials with BF vs. Trials without BF and Trials before BF vs. Trials after BF

Table 3 shows the descriptive statistics (mean, standard deviation, skewness, and kurtosis) for the maximum and mean electromyographic activity values for the trials before and after the application of BF, as well as the types of trials (without BF and with BF). Electromyographic activity is expressed in microvolts (µV).

Analyses of variance (ANOVAs) were performed to determine the differences in the mean and maximum values between types of trials (with BF vs. without BF). The data showed statistically significant differences between the types of trials, both in maximum values (F_[1,951]_ = 505.20, *p* < 0.001, η² = 0.35) and in mean values (F_[1,951]_ = 193.35, *p* < 0.001, η² = 0.17). Specifically, there were statistically significant differences between the trials with BF and those performed pre-intervention (maximum values: F_[1,714]_ = 698.60, *p* < 0.001, η² = 0.50; mean values: F_[1,714]_ = 525.79, *p* < 0.001, η² = 0.42), as well as between the trials with EMG-BF and the post-intervention trials (maximum values: F_[1,951]_ = 114.76, *p* < 0.001, η² = 0.14; mean values: F_[1,714]_ = 5.09, *p* < 0.05, η² = 0.01).

The differences between the trials before the intervention with BF and after the intervention with BF were also explored. The data showed statistically significant differences between the two both in maximum values (F_[1,469]_ = 525.45; *p* < 0.001; η² = 0.53) and in mean values (F_[1,469]_ = 577.21; *p* < 0.001; η² = 0.55).

### 3.4. Session 1 vs. Session 10

Table 4 shows the descriptive statistics (mean, standard deviation, skewness, and kurtosis) of the maximum and mean values of electromyographic activity for the trials before and after the application of BF, and also the trials with BF, both for session 1 and for session 10. Electromyographic activity is expressed in microvolts (μV).

**Figure 1:**
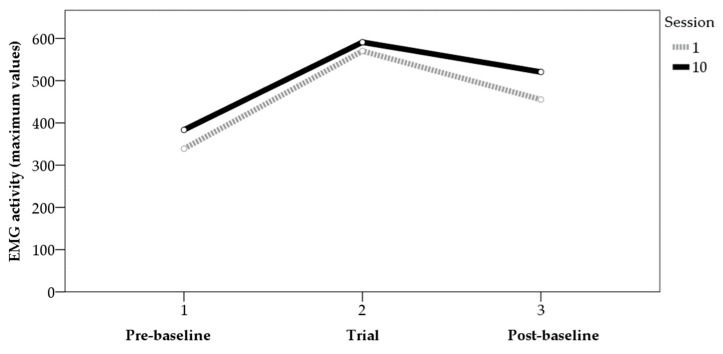
EMG activity (maximum values) pre-baseline, trial, and post-baseline (sessions 1 and 10).

**Figure 2:**
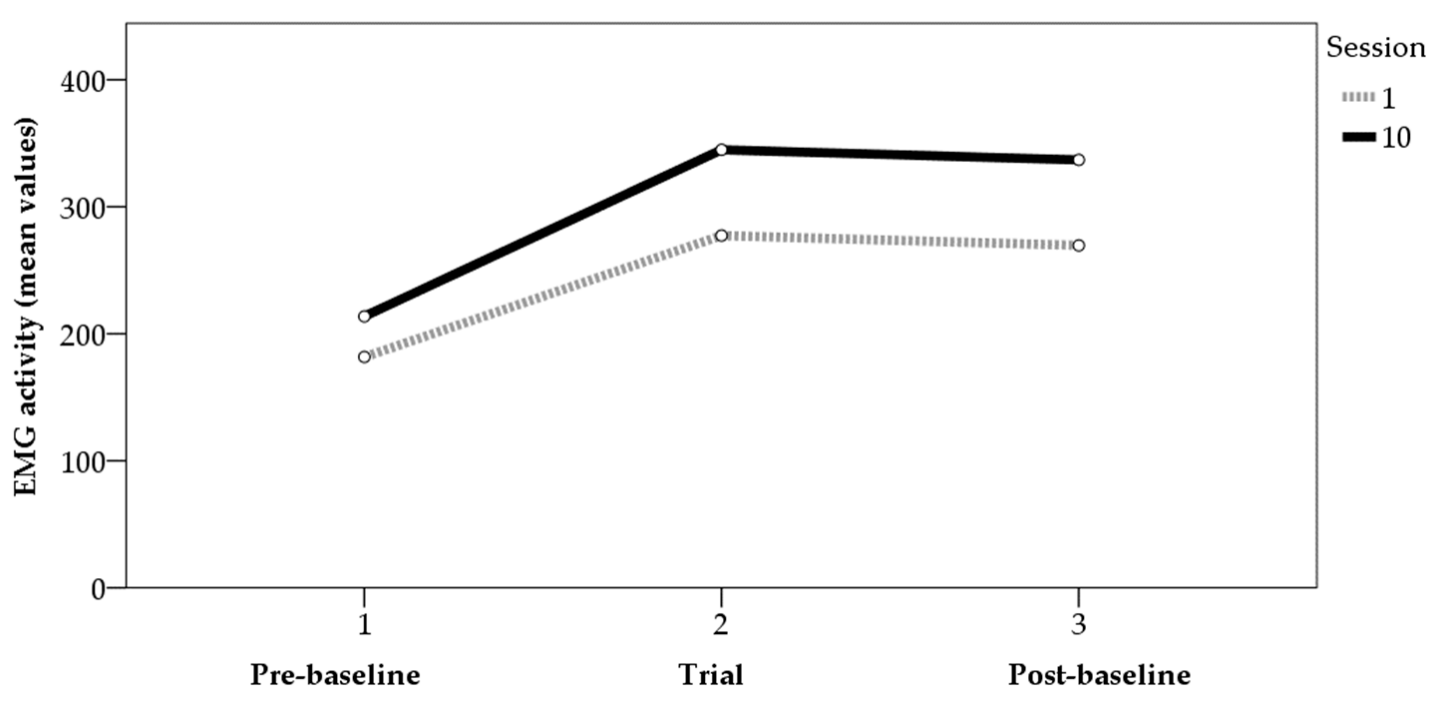
EMG activity (mean values) pre-baseline, trial, and post-baseline (sessions 1 and 10).

Analyses of variance (ANOVAs) were performed to determine the differences in average and maximum values between the first and last sessions. For the maximum values (Figure 1), the data showed statistically significant differences between the two sessions in the trials before (F_[1,31]_ = 4.15, *p* < 0.05, η² = 0.12) and after (F_[1,32]_ = 7.72, *p* < 0.01, η² = 0.19) the application of BF, though not in the trials with BF (F_[1,64]_ = 0.36, *p* > 0.05, η² = 0.01). For the mean values (Figure 2), the data showed statistically significant differences between the two sessions in the trials with BF (F_[1,64]_ = 10.53, *p* < 0.01, η² = 0.14) and in those after BF (F_[1,32]_ = 8.93, *p* < 0.01, η² = 0.22). For the values before BF, the results showed data close to significance (F_[1,31]_ = 3.51, *p* = 0.07, η² = 0.10). Figure 1 and Figure 2 show the average values of the tension generated by the contractions performed in each phase for sessions 1 and 10, for both the mean and maximum values.

## 4. Discussion

The objective of this study was to analyze the efficacy and effectiveness of an intervention with EMG-BF for control in a group of footballers who had suffered a partial meniscus tear and had undergone a meniscectomy.

Firstly, it can be seen that improvements occurred during the intervention with biofeedback, between the trials before and after that intervention, highlighting the fact that this procedure activates a learning process in which the muscle is able to behave more effectively. In addition, these gains take place in a small number of sessions and show a significant effect size, indicating the impact of that biofeedback treatment on the voluntary activity of the muscle, as has been described in previous studies [14]. The scientific literature has highlighted the clinical implications of EMG-BF, allowing the better recovery of patients with various types of muscle, bone, and ligament lesions [15,16,17,18,19,20,21]. This study is in line with those results, showing that footballers have been able to improve the contraction capacity of the vastus lateralis through the application of this technique.

These findings have great practical implications. When an injury occurs, not only does it bring the athlete’s activity to a stop, but it may cause a reduction in muscle tone and functional capacity [26,27], affecting their adaptation and subsequent performance [43,44,45]. It is therefore important to apply techniques that allow a more rapid but also more effective recovery. It has been observed that the learning that takes place with EMG-BF not only makes it easier to acquire greater awareness of the activity of the muscle, but also improves its involuntary activity [9,14]. This is of the greatest significance because it makes it possible to improve the outlook for future injuries, in a context such as soccer in which knee injuries are very common [22,23,33,37].

Regarding the data presented, it can be seen from the variance component analysis that the model presented is statistically significant and obtains an explained variance of between 67% and 75%, which reflects the effectiveness of the intervention with EMG-BF. In addition, the generalizability analysis shows that the results obtained through the intervention are reliable and can be generalized, which means that there is a high probability that the intervention and conclusions drawn are not only appropriate for the participants in this study but can be extended to other samples.

This study has some limitations. Firstly, there was no follow-up of the effects obtained: in other words, an exploration of whether the learning produced as a result of EMG-BF is retained and to what extent. It is therefore proposed that studies be conducted in which the memory of the gains obtained is assessed. Secondly, the study was carried out on one type of muscle and injury. It would be appropriate to extend it to other types of injuries to analyze whether the effects obtained through intervention with EMG-BF can be generalized to other needs for therapeutic intervention. In addition, only the behavior of the vastus lateralis has been observed, and this should be extended to other muscles involved in motor control of the knee after meniscus injuries, such as the vastus medialis and the rectus femoris. Thirdly, this study has not differentiated whether the meniscectomy was caused by injury to the medial or the lateral meniscus. In view of the possible implications of this distinction for rehabilitation, it is proposed that future studies should assess this variable. In addition to addressing these limitations, future studies should also combine this type of treatment with other therapeutic procedures to analyze the synergies established between them. A further important issue would be to determine the most appropriate treatment times for the functional rehabilitation of the athletes analyzed, observing the number of appropriate sessions for each case.

Furthermore, it would be relevant in future studies to analyze possible differences between biofeedback modalities such as ultrasound imaging, pressure biofeedback units, and electromyography. In previous research, it has been observed that different types of feedback can generate different effects depending on the protocol applied and the type of injury [57], which could enrich the knowledge developed around the recovery processes and the use of biofeedback applied in the type of injury considered in this article.

In any case, these results indicate that EMG-BF is an effective technique in neuromuscular rehabilitation after intervention with meniscectomy following partial meniscus lesions in soccer players. This has repercussions on their recovery processes and suggests that using these procedures together with other orthopedic rehabilitation interventions could help to improve the prospects for recovery of athletes who have suffered this injury.

## Figures and Tables

**Table 1:** Variance component analysis.

	Sources of Variation	Sum of Squares	Degrees of Freedom	Average Square	Random Comp.	Mixed Comp.	Corrected Comp.	%	Standard Error
**EMG-Means.**	[p]	114,837.542	9	12,759.727	−144.637	−144.637	−144.637	0	121.871
	[t]	1,093,841.16	1	1,093,841.16	3614.682	3614.682	3614.682	34.885	2977.089
	[p][t]	85,850.05	9	9538.894	307.165	307.165	307.165	2.964	135.59
	[b]	1,348,437.57	2	674,218.784	3329.438	3329.438	3329.438	32.132	2383.76
	[p][b]	149,522.483	18	8306.805	402.547	402.547	402.547	3.885	131.35
	[t][b]	0	2	0	0	0	0	0	0
	[p][t][b]	0	18	0	0	0	0	0	0
	[n]	1,047,707.54	9	116,411.949	1871.96	1871.96	1871.96	18.066	827.377
	[p][n]	337,938.074	81	4172.075	598.708	598.708	598.708	5.778	108.366
	[t][n]	1995.732	9	221.748	−3.407	−3.407	−3.407	0	3.57
	[p][t][n]	26,240.469	81	323.956	107.985	107.985	107.985	1.042	16.763
	[b][n]	5046.168	18	280.343	1.223	1.223	1.223	0.012	4.652
	[p][b][n]	41,451.469	162	255.873	127.937	127.937	127.937	1.235	14.128
	[t][b][n]	0	18	0	0	0	0	0	0
	[p][t][b][n]	0	162	0	0	0	0	0	0
**EMG-Maximum**	[p]	1,676,104.52	9	186,233.835	1367.702	1367.702	1367.702	4.142	1443.346
	[t]	5,934,252.8	1	5,934,252.8	19,517.865	19,517.865	19,517.865	59.109	16,151.388
	[p][t]	717,267.103	9	79,696.345	2618.238	2618.238	2618.238	7.929	1132.77
	[b]	1,946,405.58	2	973,202.79	4768.066	4768.066	4768.066	14.44	3440.929
	[p][b]	350,381.772	18	19,465.654	968.568	968.568	968.568	2.933	307.779
	[t][b]	0	2	0	0	0	0	0	0
	[p][t][b]	0	18	0	0	0	0	0	0
	[n]	1,404,654.19	9	156,072.688	2508.311	2508.311	2508.311	7.596	1109.286
	[p][n]	506,508.931	81	6253.197	834.949	834.949	834.949	2.529	164.499
	[t][n]	3115.654	9	346.184	−26.768	−26.768	−26.768	0	7.718
	[p][t][n]	93,086.762	81	1149.219	383.073	383.073	383.073	1.16	59.464
	[b][n]	3926.932	18	218.163	6.194	6.194	6.194	0.019	3.489
	[p][b][n]	15,274.282	162	94.286	47.143	47.143	47.143	0.143	5.206
	[t][b][n]	0	18	0	0	0	0	0	0
	[p][t][b][n]	0	162	0	0	0	0	0	0

**Table 2:** Generalizability analysis (G coefficients).

	EMG Means		EMG Maximum	
	Relative G ξρ2(δ)	Absolute G ξρ2(Δ)	Relative G ξρ2(δ)	Absolute G ξρ2(Δ)
[t] [b] [n]/[p]	0.983	0.983	0.982	0.977
[p] [t] [b]/[n]	0.989	0.966	0.996	0.987
[p] [t] [n]/[b]	0.973	0.835	0.988	0.934
[p] [b] [n]/[t]	0.968	0.759	0.875	0.483
[t] [b]/[p] [n]	0.990	0.963	0.985	0.970
[t] [n]/[p] [b]	0.981	0.817	0.983	0.911
[b] [n]/[p] [t]	0.978	0.728	0.973	0.416

**Table 3:** Descriptive statistics for maximum and mean electromyographic activity values for the trials with and without biofeedback, as well as before and after BF.

	Electromyographic Activity (μV)				
	Values	M	SD	S	K
Trials without BF (before and after)	Maximum	422.27	88.79	−0.08	−1.24
	Mean	256.89	72.59	0.18	−1.16
Pre-BF intervention trials	Maximum	357.63	54.18	1.62	5.67
	Mean	202.89	45.03	1.85	5.05
Post-BF intervention trials	Maximum	486.53	67.23	−2.22	5.19
	Mean	310.45	51.92	−0.98	2.12
Trials with BF	Maximum	578.42	122.78	0.04	−0.73
	Mean	322.98	74.12	−0.02	−0.70

Note. M = mean; SD = standard deviation; S = skewness; K = kurtosis; µV = microvolts.

**Table 4:** Descriptive statistics for maximum and mean values of electromyographic activity for trials before and after the application of BF, and also for trials with BF in sessions 1 and 10.

		Electromyographic Activity (μV)				
		Values	M	SD	S	K
Trials before BF	Session 1	Maximum	338.98	50.22	−0.89	1.89
		Mean	181.70	37.41	−1.04	0.24
	Session 10	Maximum	383.52	74.81	2.39	5.09
		Mean	213.68	59.77	2.14	4.75
Trials after BF	Session 1	Maximum	570.19	165.76	−0.04	−1.37
		Mean	277.28	92.32	0.30	−1.03
	Session 10	Maximum	591.07	101.98	0.30	−0.37
		Mean	344.85	73.31	0.20	−0.52
Trials with BF	Session 1	Maximum	455.27	86.89	−1.81	3.22
		Mean	269.57	78.14	−0.47	−1.36
	Session 10	Maximum	520.56	37.78	3.41	2.74
		Mean	336.87	47.43	1.79	5.58

Note. M = mean; SD = standard deviation; S = skewness; K = kurtosis; µV = microvolts.

## Data Availability

Data are available upon request from the authors.
